# Dual-Network Poly(vinyl alcohol)/Sodium Alginate Hydrogel Photonic Crystals Films for Visual Sensing

**DOI:** 10.3390/gels12090790

**Published:** 2026-09-01

**Authors:** Shaoqian Zhang, Xuanjun Ning, Zhangyi Qian, Yuting Zhang, Yunyan Zhang, Zixuan Zhang, Xiaoxu Zhang, Shuwen Zhang, Lishi Zhang, Cheng Chen, Donghai Lin

**Affiliations:** 1 School of Energy and Materials, Shanghai Thermophysical Properties Big Data Professional Technical Service Platform, Shanghai Engineering Research Center of Advanced Thermal Functional Materials, Shanghai Key Laboratory of Engineering Materials Application and Evaluation, Shanghai Polytechnic University, Shanghai 201209, China; 2 School of Resources and Environmental Engineering, Shanghai Polytechnic University, Shanghai 201209, China

**Keywords:** PVA, dual-network hydrogel, colloidal crystal, photonic crystal

## Abstract

Poly(vinyl alcohol) (PVA)/sodium alginate (SA) dual-network hydrogels were prepared via the freeze–thaw method. Tensile testing, electrochemical impedance spectroscopy (EIS), scanning electron microscopy (SEM), and fiber-optic spectroscopy were employed for material characterization, and multiple metal ions were screened to optimize mechanical properties and ionic conductivity. The results showed that, at PVA:SA mass ratio of 2:1, the hydrogel achieved 163% elongation and 0.18 MPa tensile strength. Ca^2+^ crosslinking formed an enhanced structure with mechanical properties of 170% elongation and 0.21 MPa strength, and ionic conductivity (0.69 S/m). Combined with colloidal photonic crystal (PC) templates, the PVA/SA-PC film exhibited an inverted opal structure, showing sensitive color response (green to red) and diffraction red-shift toward Ca^2+^. The conductive film could power a small bulb, demonstrating potential for portable visual sensing applications.

## 1. Introduction

With the rapid development of intelligent materials, electronic-skin-oriented flexible devices have been progressively engineered and have gained growing attention in human-behavior monitoring, health monitoring, and ion-detection fields. As a critical interface between humans and electronic devices, electronic skin mimics the tactile and perceptual functionalities of human skin, enabling continuous monitoring of physiological signals such as body motion, pulse, respiration, and temperature [1,2,3]. This capability holds tremendous promise for wearable healthcare, human–machine interfaces, and smart robotics [4,5]. As the most commonly used substrate in flexible sensors, hydrogels feature a special three-dimensional network structure, enabling them to load various functional materials such as highly conductive polymers, high-strength materials, and high-viscosity natural substances [6]. The highly hydrated and soft nature of hydrogels ensures comfortable interfacing with human tissues, while their porous architecture facilitates the incorporation of functional nanomaterials to impart electrical conductivity, mechanical reinforcement, or stimuli-responsive behavior [7,8,9]. This has made hydrogels a research hotspot in recent years and been applied in diverse scenarios. For instance, conductive hydrogels have been widely employed in strain sensors, pressure sensors, and biosensors, demonstrating excellent sensitivity and stability [10,11]. However, most hydrogel-based flexible sensors cannot intuitively display changes; they only collect signals generated by internal variations (e.g., ion transport and structural deformation). These signals usually require additional instruments for detection, which greatly increases the cost and inconvenience of use. Moreover, the signal processing and data transmission introduce inevitable time delays, limiting real-time responsiveness in scenarios requiring instantaneous feedback [12,13]. Among various sensing targets, rapid ion detection, especially calcium ion monitoring, carries practical value for water-quality screening, wearable wound-exudate analysis and point-of-care testing. Conventional analytical instruments are accurate but bulky, so low-cost, visual on-site ion-sensing approaches are highly desirable.

Visual sensing represents an elegant solution to the aforementioned limitations, as it converts imperceptible stimuli into directly observable color changes, eliminating the need for complex instrumentation and enabling instantaneous user interpretation [14,15]. Visual sensors based on hydrogel photonic crystals are an important branch of electronic skin. They incorporate structurally colored photonic crystals into the hydrogel network, and the deformation of the hydrogel network changes the arrangement of photonic crystals [16]. Photonic crystals are periodic dielectric nanostructures that manipulate the propagation of light through Bragg diffraction, generating vivid structural colors without pigments or dyes [17,18]. Unlike conventional colorants that rely on light absorption and are prone to photobleaching, structural colors originate from physical interactions between light and ordered nanostructures, offering advantages of high brightness, non-fading, and environmental friendliness [19,20]. As indicated by Bragg’s law, variations in lattice spacing and refractive index can shift the diffraction wavelength of photonic crystals [21]. Specifically, when the hydrogel matrix swells or shrinks in response to external stimuli such as mechanical force, temperature change, pH variation, or ion binding, the embedded photonic crystal lattice undergoes corresponding dimensional changes, resulting in a perceptible shift in the reflected color [22,23]. Hydrogels act as a stable carrier for photonic crystals under moderate external stimuli. Inspired by the combination of the optical properties of photonic crystals and flexible hydrogels, this composite has been applied in anti-counterfeiting, colorimetric sensing, photocatalysis, and other fields [24,25,26,27,28,29,30,31,32].

**Scheme 1:**
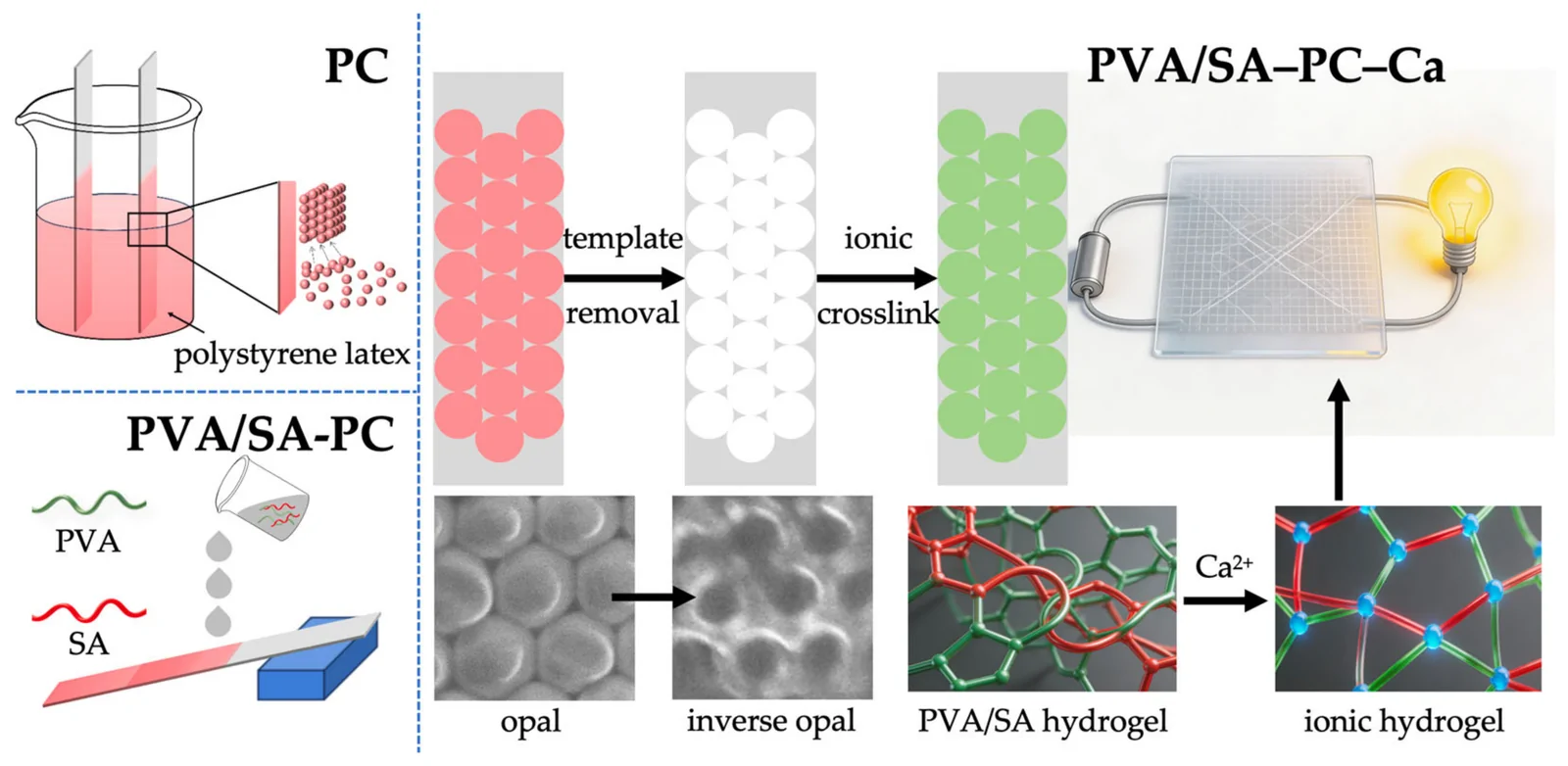
Schematic illustration of PVA/SA hydrogel photonic crystal film preparation.

In this work, to address the aforementioned challenges, we designed a dual-network hydrogel system combining poly(vinyl alcohol) (PVA) with sodium alginate (SA). PVA was selected for its excellent biocompatibility, non-toxicity, and tunable mechanical properties achieved through cyclic freeze–thaw processing, while SA was chosen for its unique ion-responsive properties and ability to form specific crosslinked structures with multivalent cations [6,33,34]. Mechanical and conductive properties of PVA/SA dual-network hydrogels were tested to determine the optimal mass ratio and the most suitable metal ion for conductivity enhancement. The systematic optimization process involved evaluating tensile strength, elongation at break, compressive strength, and ionic conductivity across different formulations, ensuring a comprehensive understanding of the structure-property relationships. The hydrogels were then combined with photonic crystals to fabricate PVA/SA–PC films, whose mechanical and conductive properties were characterized. The photonic crystal templates were prepared via vertical deposition of monodisperse polystyrene nanospheres, followed by infiltration of the optimized PVA/SA precursor solution and subsequent template removal to create an inverse opal structure. By virtue of the inverse-opal structure within films, we fabricated visual sensors for ion detection, which realize real-time monitoring and instant feedback through visible color variation. Specifically, the inverse opal architecture provides fast ion transport channels and enhanced the interaction between target ions and the hydrogel matrix, thereby amplifying the optical response sensitivity. Moreover, the conductive PVA/SA–PC films could light an LED bulb in an electric circuit, demonstrating the dual-mode sensing capability that combines visual and electrical signal output. The preparation route was displayed in Scheme 1.

## 2. Results and Discussion

### 2.1. Characterization of PVA/SA Hydrogels

**Figure 1:**
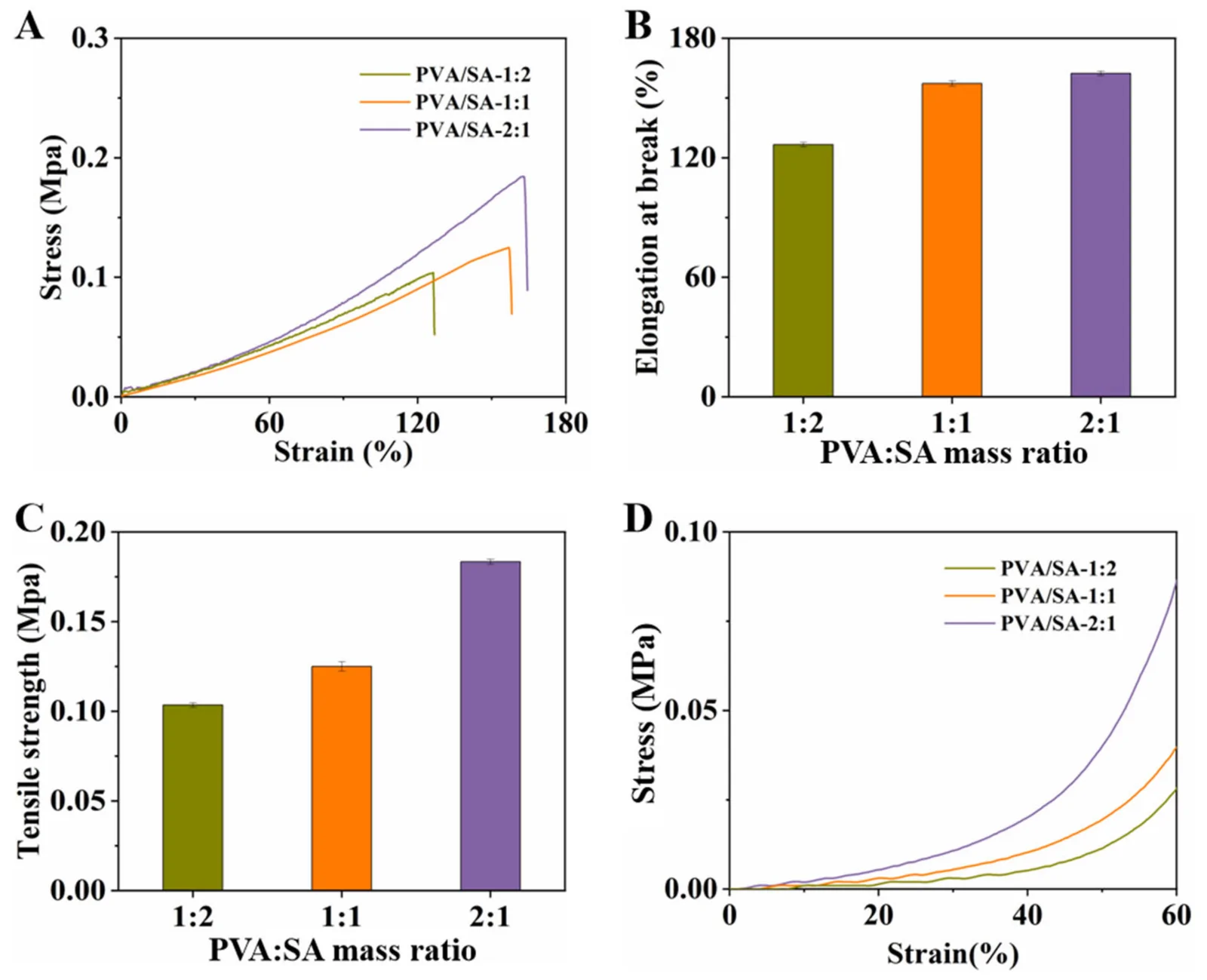
(**A**) Tensile stress–strain curves at different mass ratios; (**B**) Elongation at break at different mass ratios; (**C**) Tensile strength at different mass ratios; (**D**) Compressive stress–strain curves at different mass ratios. (*n* ≥ 3).

The mechanical properties of the prepared hydrogels were systematically evaluated by tensile and compression tests. The mass ratio of PVA and SA was verified to be a critical factor governing the overall mechanical performance, where PVA mainly contributed high strength, while SA provided good toughness. Tensile behaviors of PVA/SA hydrogels at different mass ratios are shown in Figure 1A–C. At a PVA:SA mass ratio of 1:2, the elongation at break of the hydrogel was 125%. As the PVA content increased, the network became denser with enhanced energy dissipation capability. At a mass ratio of 2:1, the elongation at break reached 163%, and the tensile strength increased from 0.11 MPa to 0.18 MPa. These results confirm that adjusting the PVA/SA ratio improves tensile performance while maintaining shape stability through enhanced network integrity.

Compression tests (Figure 1D) showed that at 60% strain, the compressive strength was only 0.026 MPa at PVA:SA = 1:2, because excessive SA made the hydrogel overly soft and prone to deformation. With increasing PVA content, the compressive strength increased, reaching a maximum of 0.08 MPa at PVA:SA = 2:1. After cyclic freezing–thawing, the solution underwent gelation, forming a PVA crystalline network, while a small amount of SA chains crosslinked with the PVA network through hydrogen bonds. The mechanical strength was dominated by the PVA network. Therefore, the PVA:SA mass ratio of 2:1 was chosen for subsequent experiments.

**Figure 2:**
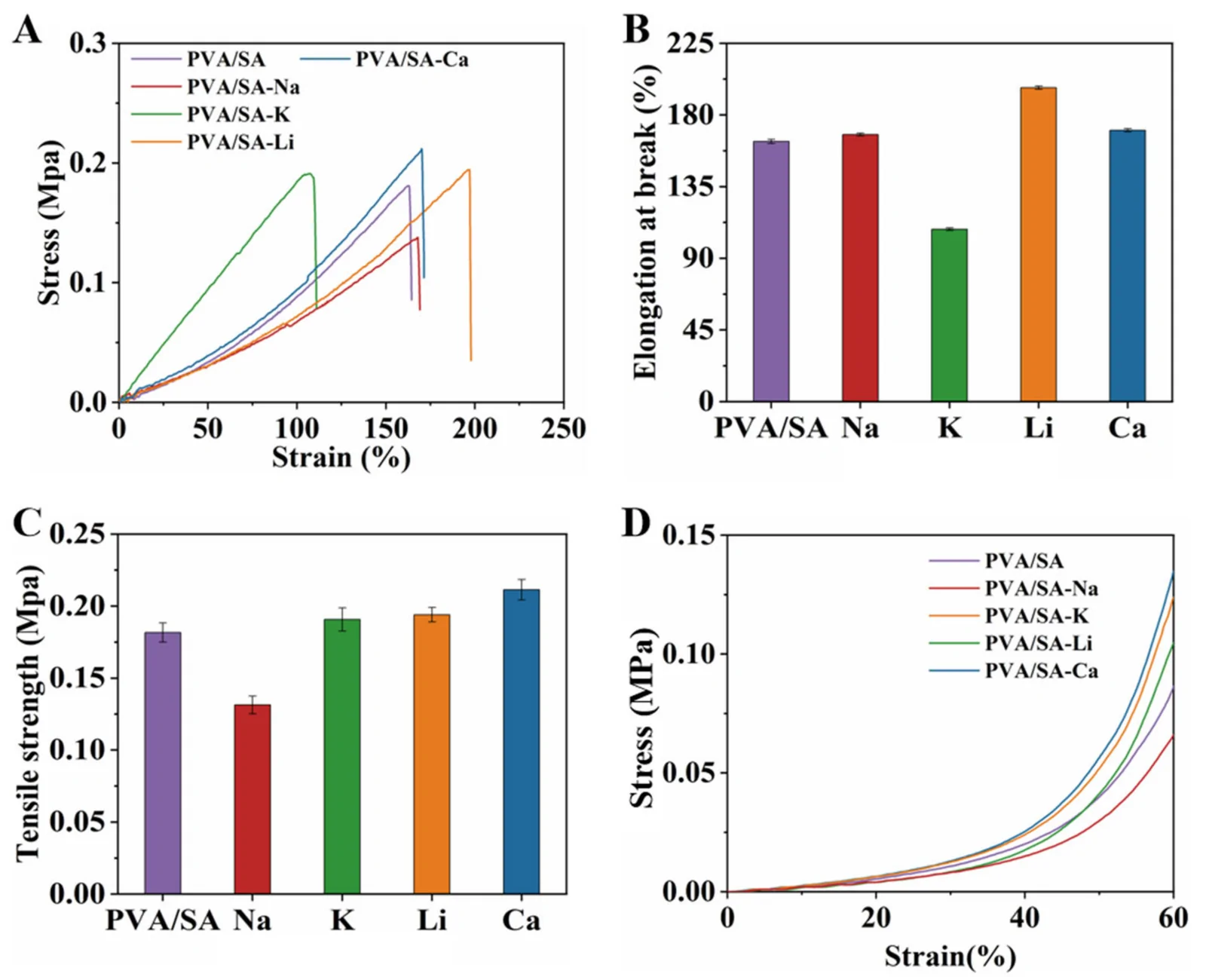
(**A**) Tensile stress–strain curves of PVA/SA hydrogels with different metal ions; (**B**) Elongation at break with different ions; (**C**) Tensile strength with different ions; (**D**) Compressive stress–strain curves of PVA/SA hydrogels with different ions. (*n* ≥ 3).

The prepared PVA/SA hydrogels were soaked in various metal ion solutions to investigate ion effects on mechanical properties (Figure 2A–C). Compared with neat hydrogels, samples soaked with Na^+^, K^+^, Li^+^, and Ca^2+^ showed improved elongation at break or tensile strength. Notably, Ca^2+^ complexed with the G-blocks of SA chains to form an enhanced structure, constructing an ionically crosslinked network intertwined with the PVA network. This dual-network structure significantly enhanced mechanical performance. Although Li^+^-incorporated hydrogels exhibited higher elongation than Ca^2+^-loaded ones, their tensile strength was lower, likely due to incomplete ionic crosslinking and weak energy dissipation. With Ca^2+^, the elongation at break reached 170% and tensile strength reached 0.21 MPa.

Compression tests (Figure 2D) revealed that Ca^2+^-loaded hydrogels displayed higher stress at every strain level than other ion-containing samples. The dual-network composed of Ca^2+^–SA crosslinks and PVA crystalline domains, strengthened by hydrogen bonding, provided excellent energy dissipation under mechanical stress.

**Figure 3:**
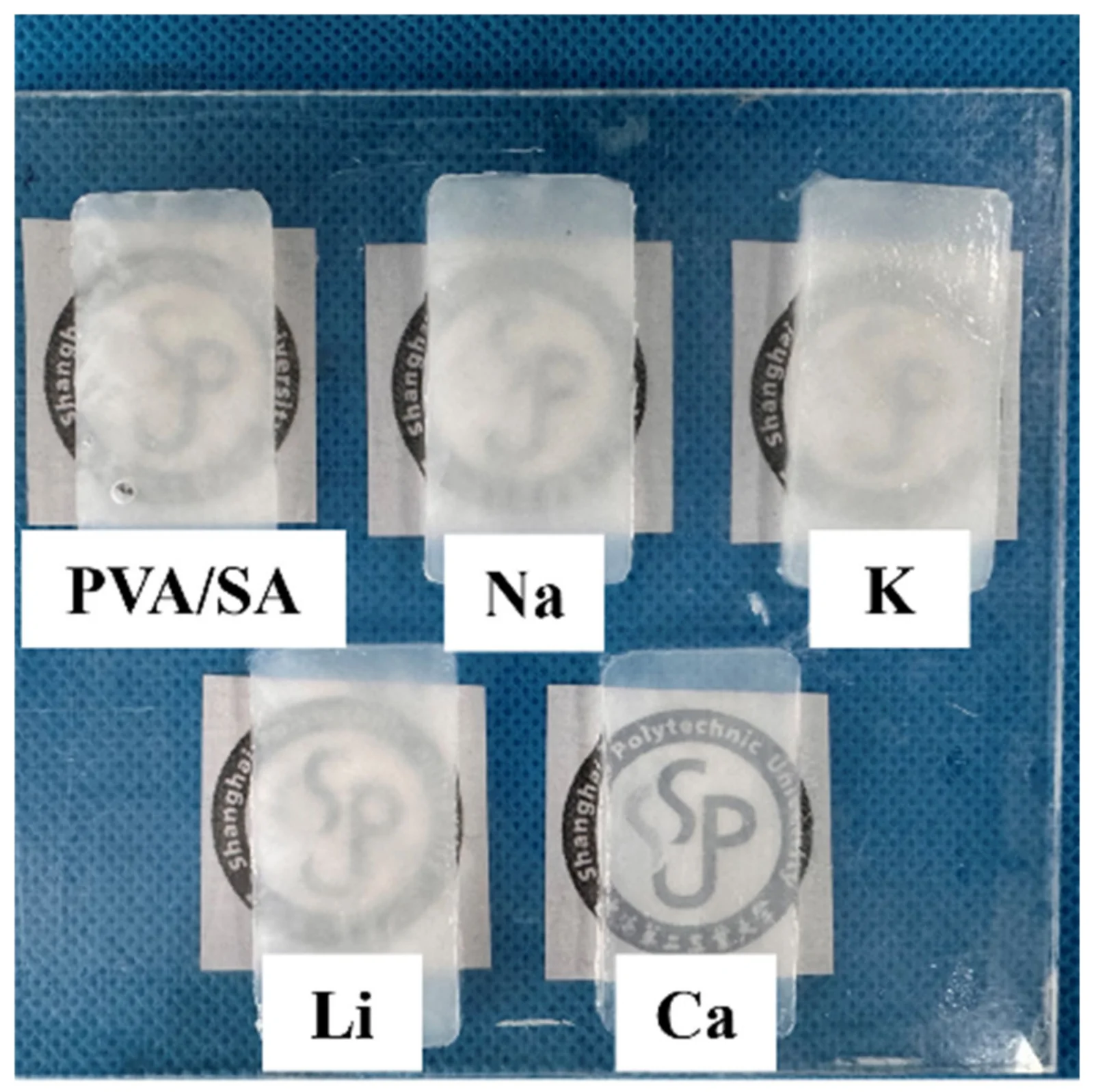
Optical images of PVA/SA hydrogels incorporated with different metal ions.

The optical appearance of hydrogels after ion soaking is shown in Figure 3. Neat PVA/SA hydrogels and those with Na^+^, K^+^, and Li^+^ were opaque with poor transmittance, due to weak ionic crosslinking. In contrast, Ca^2+^-loaded hydrogels were highly transparent, clearly revealing the underlying pattern, indicating that Ca^2+^ optimized the crosslinked network structure.

**Figure 4:**
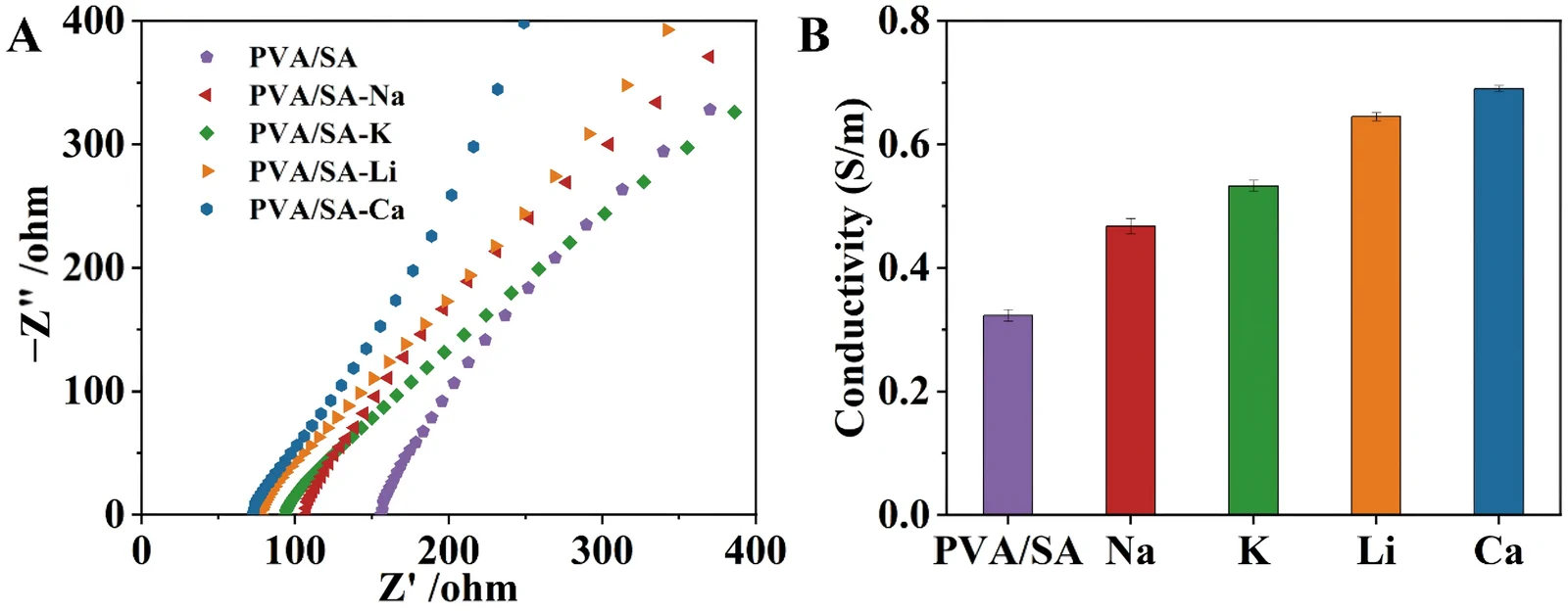
(**A**) EIS plots of PVA/SA hydrogels with different metal ions; (**B**) Ionic conductivity of PVA/SA hydrogels with different metal ions. (*n* ≥ 3).

After soaking in metal ion solutions, PVA/SA hydrogels contained abundant free mobile ions, and SA chains were ionically crosslinked by Ca^2+^, endowing the hydrogels with noticeable conductivity. Conductivity was analyzed via electrochemical impedance spectroscopy (EIS) (Figure 4A), and ionic conductivity was calculated (Figure 4B). Neat PVA/SA hydrogels had high impedance, with a conductivity of 0.32 S/m. After ion soaking, conductivity improved, reaching a maximum of 0.69 S/m when soaking in CaCl_2_ solution. Thus, Ca^2+^ provided the best conductivity. Based on comprehensive mechanical and conductive tests, Ca^2+^ (from CaCl_2_) was selected for further studies.

### 2.2. Synthesis and Morphology of Polystyrene Nanospheres

**Figure 5:**
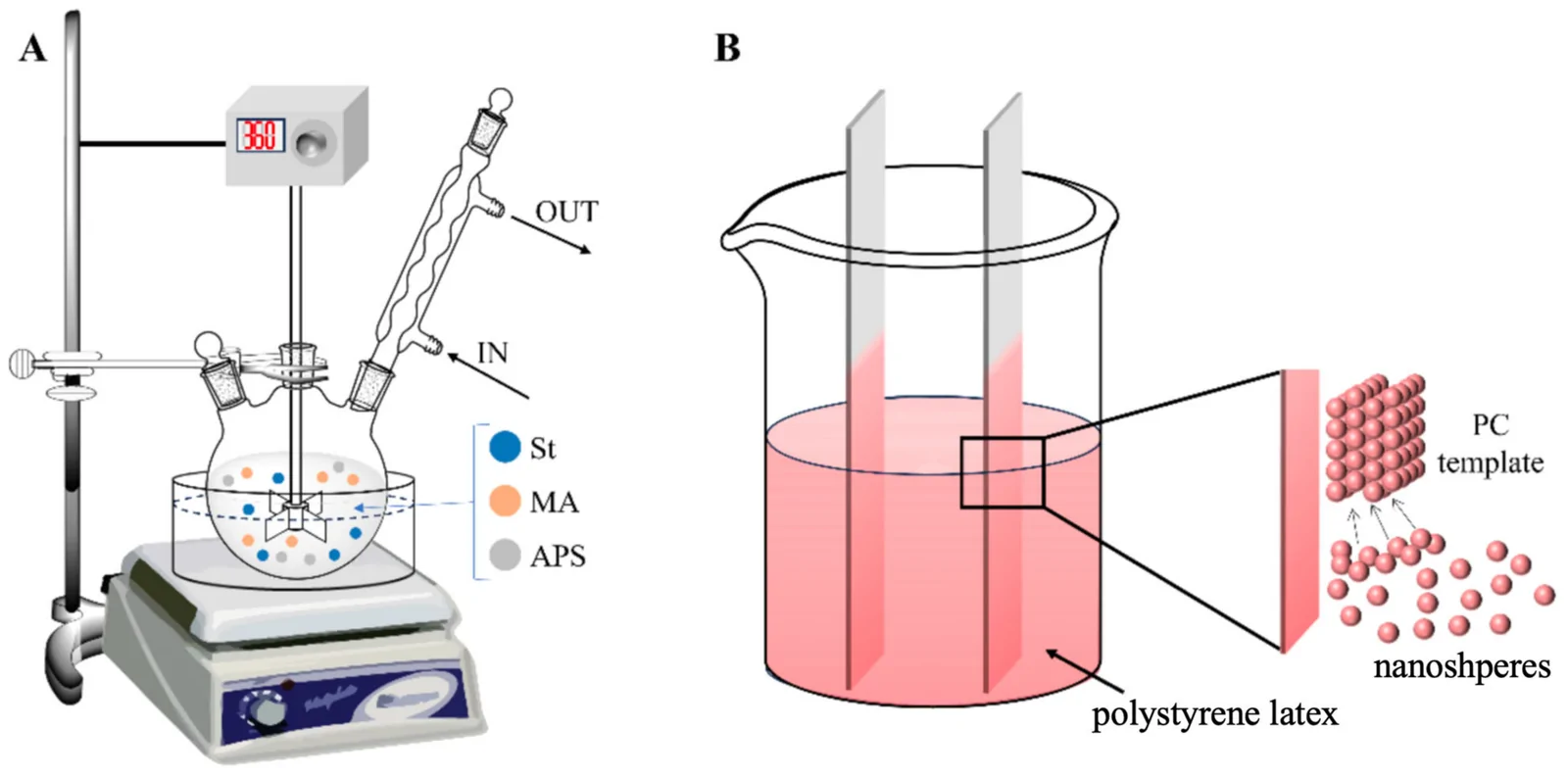
(**A**) Schematic illustration of the synthesis setup for polystyrene nanosphere emulsion; (**B**) Schematic of self-assembled PC template from polystyrene nanospheres.

All polystyrene nanospheres were lab-synthesized via non-boiling emulsion polymerization. Monodisperse polystyrene nanospheres with a diameter of ~252 nm were fabricated at a constant oil-bath temperature of 98 °C with fixed stirring to ensure uniform reaction. The target particle size was controlled by adjusting the polymerization time initiated by APS (Figure 5A). In recent studies, photonic crystals are usually assembled from monodisperse colloidal nanospheres, which self-organize into periodic array structures. This method features simple preparation, low cost, and high structural order. During self-assembly, nanospheres undergo diffusion, rearrangement, and ordered packing to form a face-centered cubic lattice. Colloidal crystal assembly methods include physical confinement, template-assisted, and vertical deposition.

To observe the microstructure, PS nanospheres were self-assembled into PC templates via vertical deposition. With solvent evaporation, colloidal spheres packed into a highly ordered array on the modified glass surface under capillary forces (Figure 5B).

**Figure 6:**
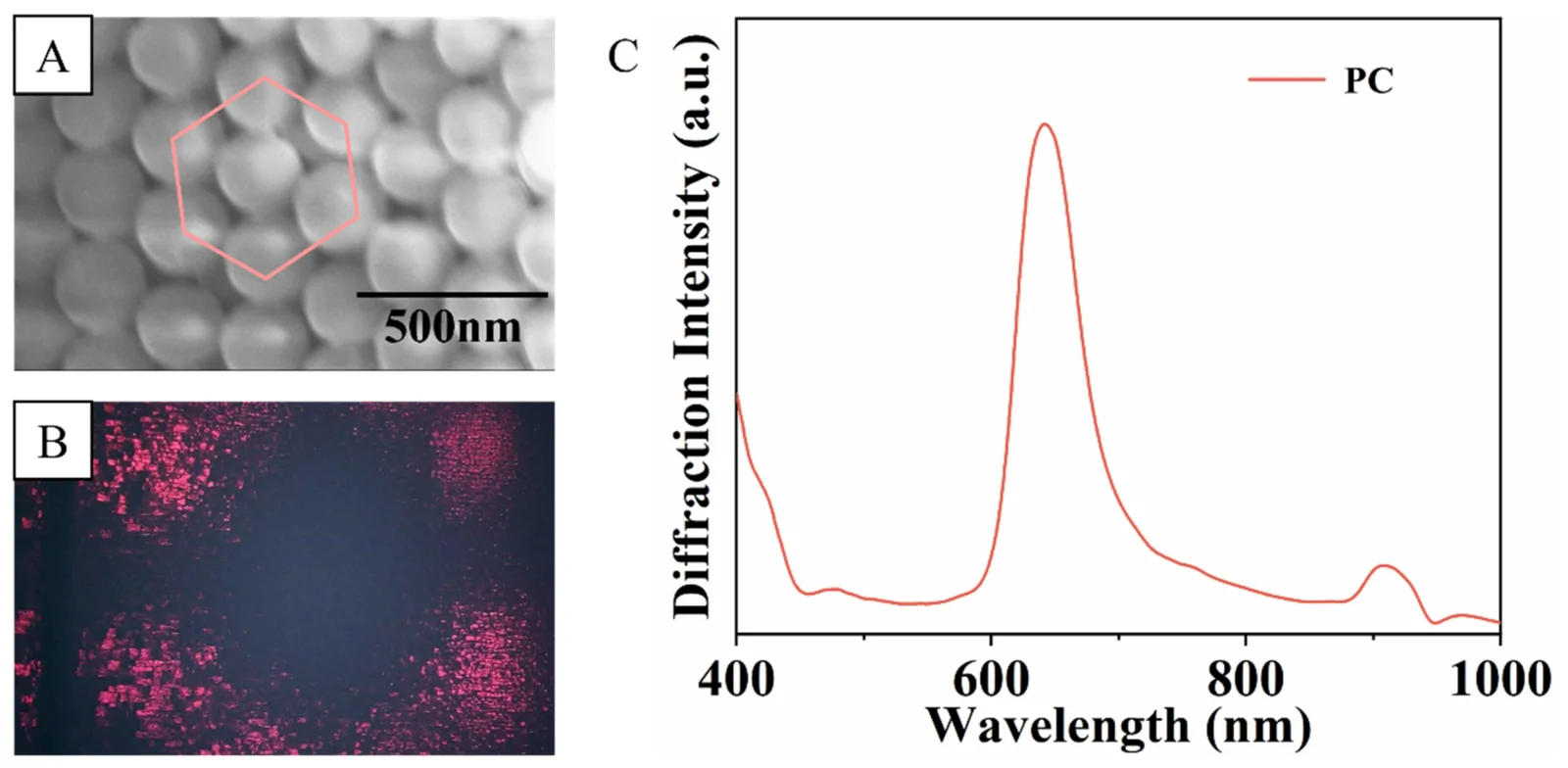
(**A**) SEM image of 252 nm polystyrene nanospheres; (**B**) Structural color of 252 nm polystyrene nanospheres; (**C**) Reflection spectrum of the PC template.

Polystyrene nanospheres were transferred onto conductive tape, sputter-coated with gold, and observed by SEM. As shown in Figure 6A, the nanospheres were uniform in size and arranged in a hexagonal lattice. Under an optical microscope, 252 nm polystyrene nanospheres appeared bright red structural color (Figure 6B). Fiber optic spectroscopy revealed a single reflection peak at approximately 650 nm (Figure 6C), confirming the high uniformity and regularity of the PC array.

### 2.3. Structural Analysis of PVA/SA–PC

**Figure 7:**
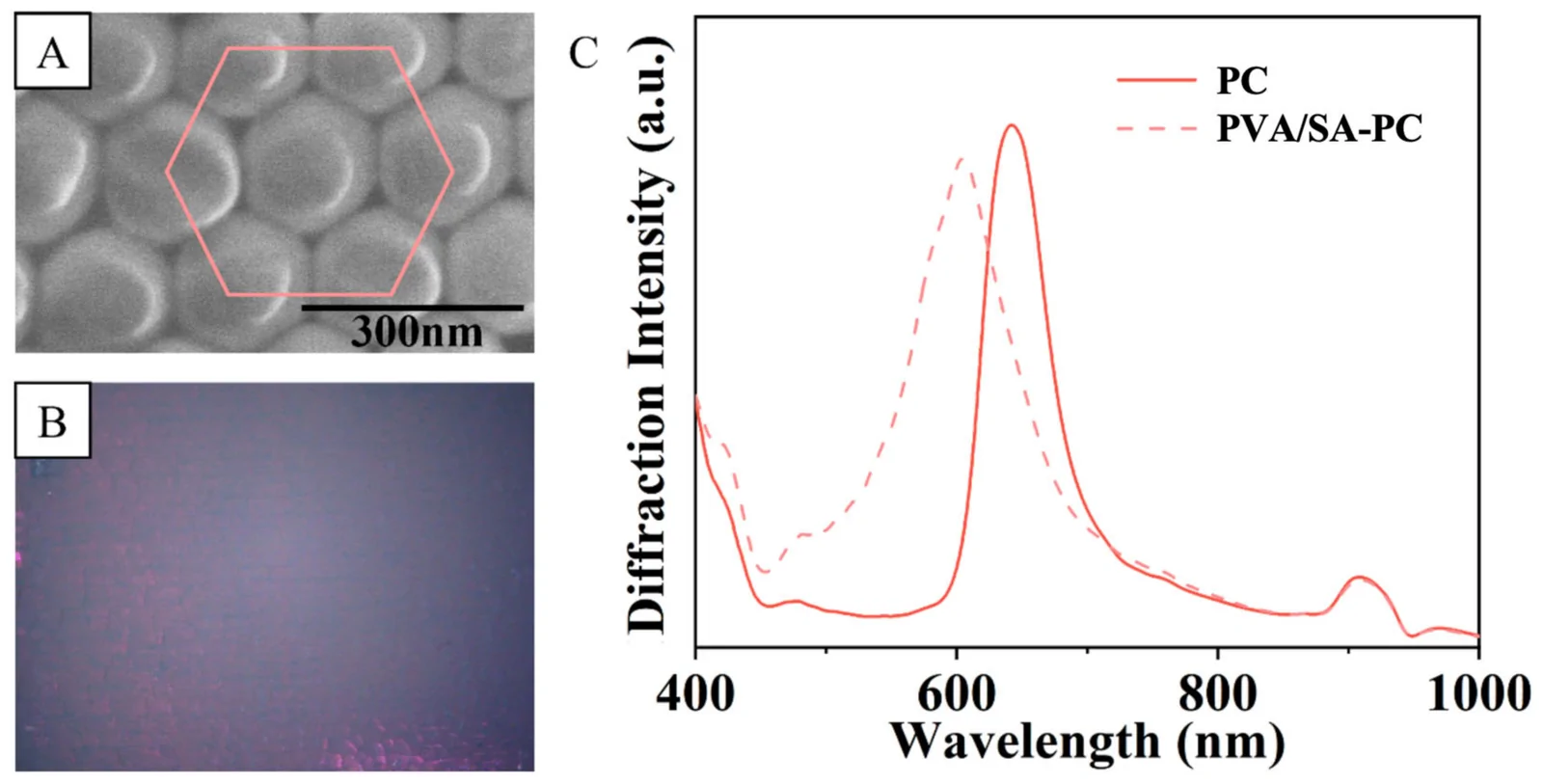
(**A**) SEM image of PVA/SA–PC film; (**B**) Structural color of PVA/SA–PC film; (**C**) Reflection spectrum of PVA/SA–PC film.

After the PVA/SA hydrogel precursor fully infiltrated and filled the voids of the PC template, the original hexagonal structure of polystyrene nanospheres was well maintained, while the inter-particle spacing was slightly reduced (Figure 7A). This structural change was attributed to the internal electric field generated by ionic SA hydrogel, which compressed the ordered polystyrene nanosphere assembly to form a tighter packing structure, endowing the composite film with a uniform and stable structural color (Figure 7B). The optical diffraction behavior of the PC structure conforms to Bragg’s diffraction law:

According to Bragg’s diffraction law:

(1) $$\lambda_{0}=2n_{a}d\sin\theta$$

where $\lambda_{0}$ is diffraction peak wavelength, *n*_a_ is effective refractive index, θ is incident angle, and *d* is interplanar spacing of the crystal.

During measurement, the fiber probe was perpendicular to the sample (θ = 90°), thus the diffraction wavelength of PVA/SA–PC films was mainly depended on *n*_a_ and *d*. SEM observations showed that the compacted nanospheres reduced *d*, Although PVA (*n*_PVA_ = 1.52) and SA (*n*_SA_ = 1.52) hydrogel slightly increase the *n*_a_ of the system (relative to air, *n*_air_ = 1), the system still exhibits a blue shift under the overall influence of *d* and *n*_a_, which was observed by the diffraction spectrum (Figure 7C).

### 2.4. Mechanical Properties of PVA/SA–PC

**Figure 8:**
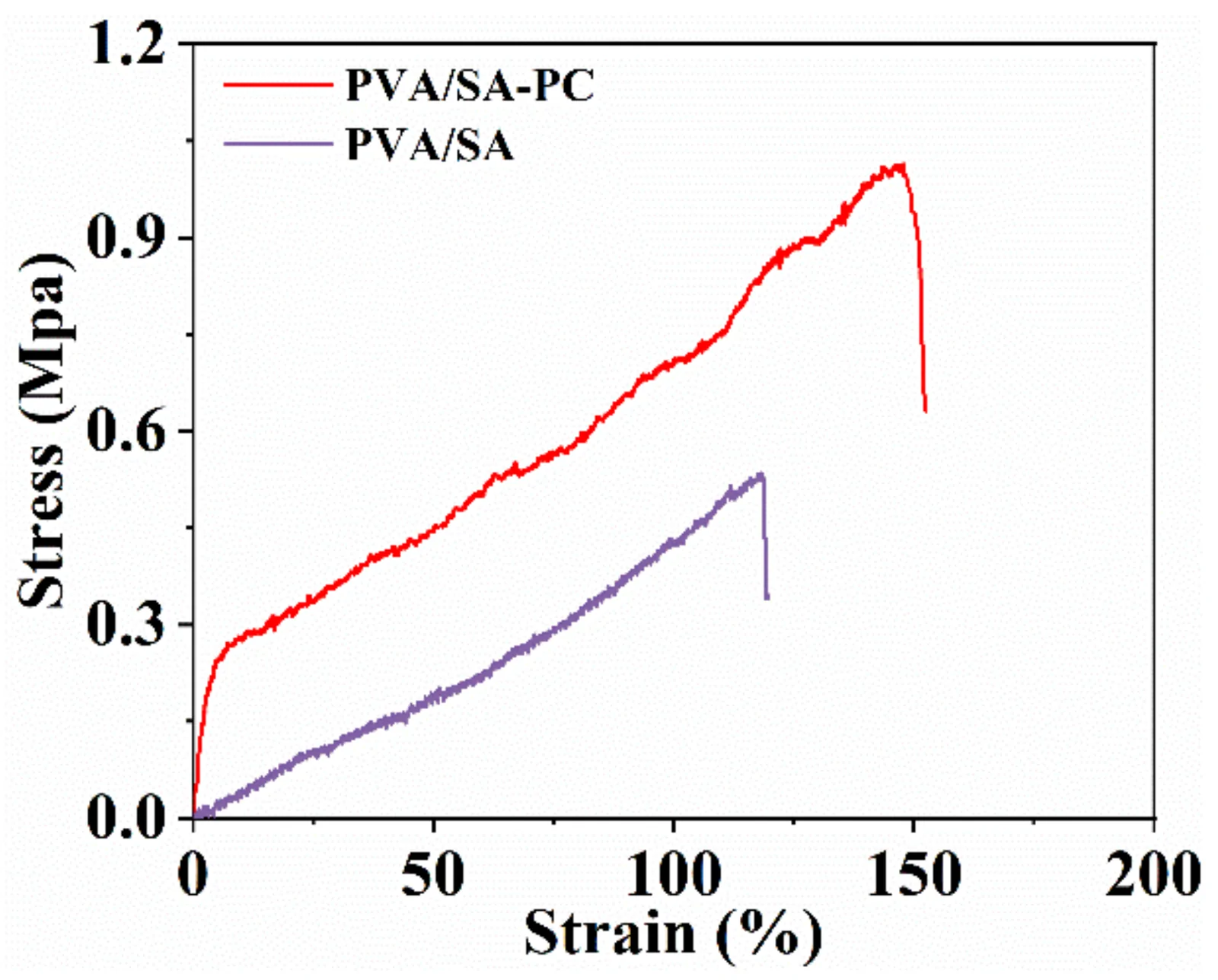
Stress–strain curves of PVA/SA and PVA/SA–PC films.

For consistent testing, PVA/SA precursor solutions were cast into polytetrafluoroethylene molds and dried at 70 °C to form films. Both PVA/SA and PVA/SA–PC films were rigid in the dry state. Therefore, films were soaked in deionized water for 30 s to soften before tensile testing. As shown in Figure 8, neat PVA/SA films showed a maximum elongation of 117% and tensile strength of 0.52 MPa. After integrating the PC template, the elongation increased to 147.5% and tensile strength rose to 1.01 MPa. In the dehydrated state, the ductility of PVA/SA films was restricted by crystalline chains and lattice units. The PC template acted as a reinforcing filler, the ordered colloidal spheres were uniformly dispersed in the polymer matrix, stabilizing the macromolecular structure and strengthening intermolecular interactions. Thus, the PC layer significantly improved elongation at break, network compactness, and structural rigidity, leading to clearly enhanced stress–strain performance.

### 2.5. Conductive Properties of PVA/SA–PC

The PC template method is widely used to fabricate inverse opal films. The typical procedure includes three steps: (1) Preparation of the PC template; (2) Infiltration of hydrogel precursor into the template interstices; and (3) Removal of the template using an organic solvent (e.g., toluene).

A highly ordered PC template is critical to ensure the integrity of the inverse opal structure; defects would destroy the periodic array. Proper concentration of monodisperse polystyrene nanospheres and drying temperature produced PC templates with uniform color and morphology.

**Figure 9:**
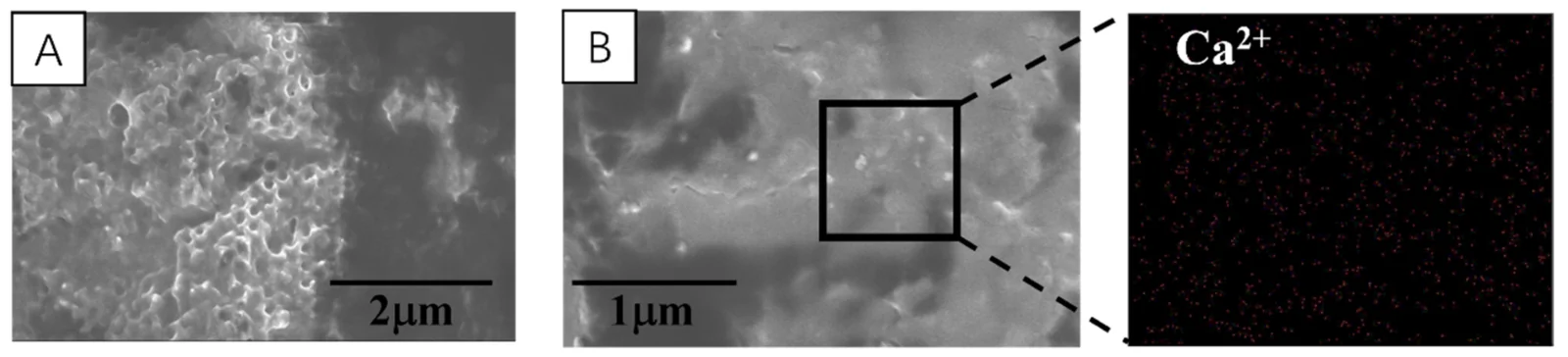
(**A**) SEM image of the inverse opal structure of PVA/SA–PC film; (**B**) Local magnified image and EDX mapping of PVA/SA–PC film after soaking.

To enhance binding capacity, the films were immersed in 5 mL of toluene for 48 h to etch away the polystyrene nanopheres, yielding a porous inverse opal structure denoted as PVA/SA–PCA. The inverse opal exhibited a well-defined honeycomb pore structure (Figure 9A), which provided fast transport channels for ions and enabled conductive behavior under applied voltage.

The PVA/SA–PCA films were soaked in calcium chloride solution for 5 min and wiped dry to obtain PVA/SA–PCA–Ca. Part of participated in ionic crosslinking of SA chains, densifying the hydrogel network; the rest existed as free mobile ions inside the inverse opal pores. EDX mapping confirmed the uniform distribution of throughout the film (Figure 9B).

**Figure 10:**
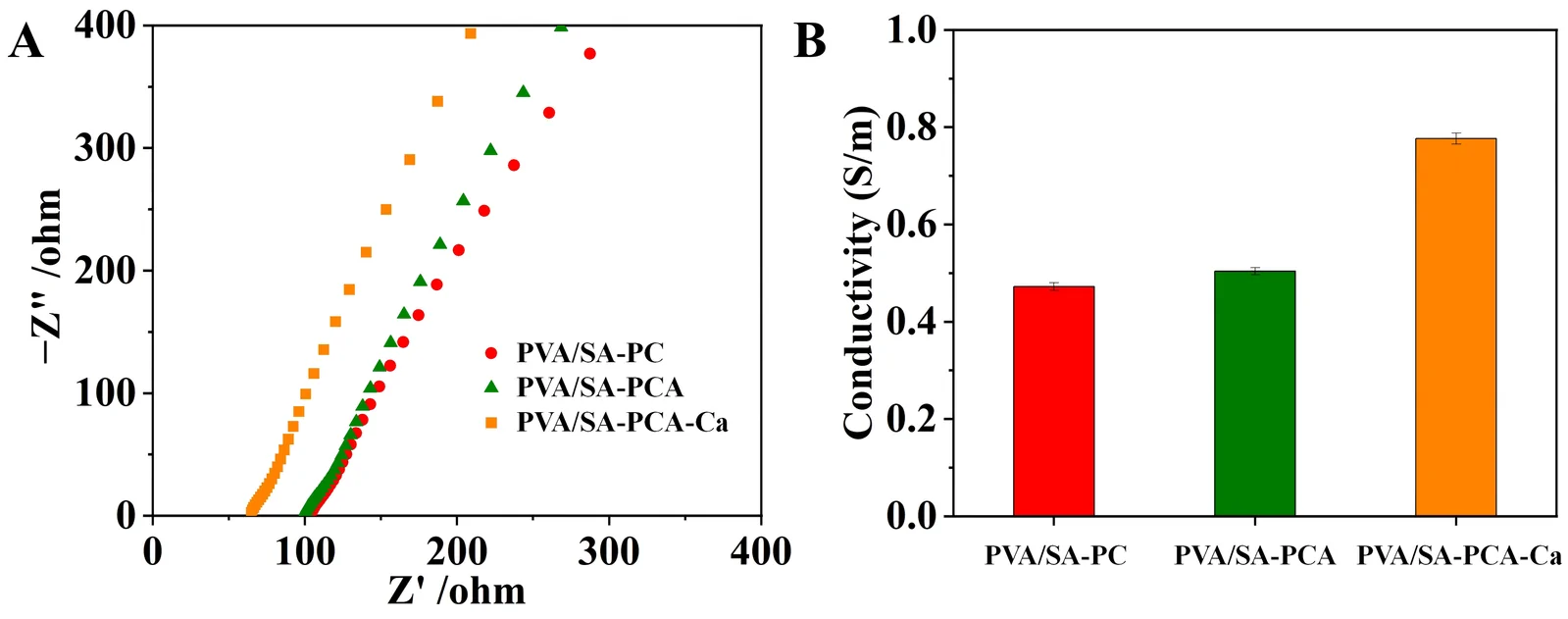
(**A**) Impedance plots of PVA/SA–PC, PVA/SA–PCA, and PVA/SA–PCA–Ca films; (**B**) Conductivity of PVA/SA–PC, PVA/SA–PCA, and PVA/SA–PCA–Ca films. (*n* ≥ 3).

Meanwhile, the PVA/SA–PCA–Ca film was endowed with a certain conductivity by soaking in a calcium chloride solution. Therefore, an electrochemical workstation was used to characterize its electrical conductivity (Figure 10A).The PVA/SA–PC film exhibited a high impedance due to the closely packed internal nanospheres, which hindered ion transport. After the nanospheres were removed with toluene, the inverse opal pores facilitated ion migration. However, the number of free ions inside the hydrogel film was low at this stage, resulting in no significant improvement in conductivity (Figure 10B).

### 2.6. Visual Sensing Performance of PVA/SA–PC

Traditional sensors convert stimuli into electrical signals that require processing equipment for readout, leading to delayed feedback and complicated operation without direct visualization. This inevitably causes delayed signal feedback and complicated operation, and cannot realize intuitive visual detection. In view of this, the as-prepared PVA/SA–PC films were tested for visual sensing toward solution ions.

**Figure 11:**
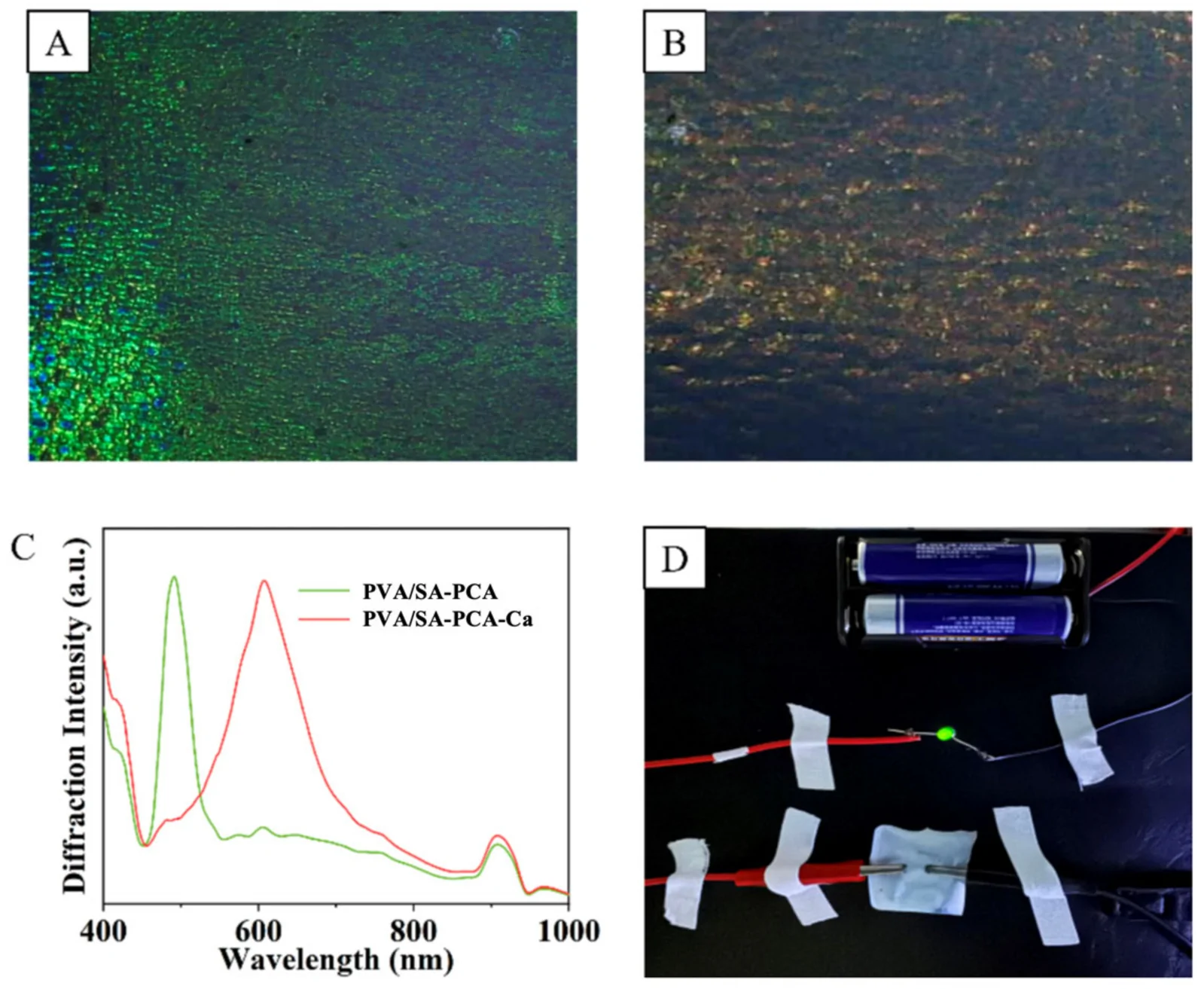
(**A**) Structural color of the PVA/SA–PC inverse opal film; (**B**) Structural color after exposure to Ca^2+^; (**C**) Diffraction spectra before and after introduction; and (**D**) PVA/SA–PCA–Ca film lighting an LED bulb in a circuit.

Ion-sensing performance is displayed in Figure 11A,B. The original PVA/SA–PCA hydrogel film was green. Upon immersion in 0.1 M CaCl_2_ solution, a visible color shift from green to reddish-yellow could be observed by the naked eye within approximately 10~30 s; the diffraction peak stabilized within 2~5 min. Diffraction spectra (Figure 11C) showed that Ca^2+^ increased the effective refractive index and enlarged the lattice spacing by occupying inverse opal pores, resulting in a red shift in the diffraction peak. Correspondingly, the structural color changed from green to reddish-yellow, realizing visual ion detection. Benefiting from structural color-based output, our system achieves rapid visual readout within several minutes. On the other hand, conventional electrochemical sensors usually involve multi-step signal-processing workflows that may also take several minutes. Both strategies possess their respective merits. Our approach illustrates one desirable feature offered by visual-sensing strategies, and the combination of these two sensing modalities could be explored in future work. When connected in a circuit (Figure 11D), the conductive PVA/SA–PCA–Ca film successfully lit an LED bulb, verifying its conductive capability.

It is worth noting that the current work focused on the proof-of-concept demonstration of qualitative visual detection toward Ca^2+^. Other cations such as Na^+^, K^+^, Li^+^, Mg^2+^, Ba^2+^, and Fe^3+^ may interact differently with alginate G-blocks due to differences in ionic radius and charge density, which could trigger distinct network shrinkage or swelling and produce varied color responses. Systematic investigation of ion selectivity, cross-sensitivity, underlying ion-exchange mechanisms, as well as quantitative sensing parameters including sensitivity, detection limit, repeatability, and the quantitative correlation between spectral shift and Ca^2+^ concentration will be pursued in our future studies. Nonetheless, the present material shows promise for portable semi-quantitative Ca^2+^ visual detection and is particularly suited for scenarios demanding instant visual feedback, such as (1) rapid on-site screening of Ca^2+^ in water quality monitoring; (2) real-time tracking of ionic fluctuations in wound exudate through wearable devices; and (3) visual-electrical dual-mode sensing nodes in flexible electronic skins. The primary advantage of this approach is the elimination of external signal-processing equipment, enabling rapid detection via direct color change. Practical validation using complex samples and further performance optimization are still required before implementation.

## 3. Conclusions

In this work, high performance PVA/SA dual-network hydrogels were successfully prepared, and their mechanical and conductive properties were systematically characterized to determine the optimal formulation. The PVA crystalline network and ionic crosslinked SA network endows the hydrogel with excellent energy dissipation capacity and structural stability. At a PVA:SA mass ratio of 2:1, the hydrogel achieved an elongation at break of 163% and a tensile strength of 0.18 MPa. After introducing Ca^2+^, the ionic crosslinking effect further optimized the hydrogel network structure, the elongation at break increased to 170%, tensile strength reached 0.21 MPa, and conductivity was significantly improved. Furthermore, as combined with self-assembled PC templates, the hydrogel exhibited further enhanced mechanical performance. The PVA/SA–PC film showed sensitive visual response to Ca^2+^ with distinct color variation and diffraction peak shift. Meanwhile, the conductive film could drive an LED bulb in a circuit. All the above results demonstrate that the fabricated PVA/SA–PC inverse opal film possesses promising application prospects in portable and intuitive visual sensing devices.

## 4. Materials and Methods

### 4.1. Materials and Reagents

Anhydrous calcium chloride (AR), ammonium persulfate (APS, AR, ≥98.0%), methacrylic acid (MA, 98%), Poly(vinyl alcohol) (PVA, 99% hydrolyzed, DP = 1750 ± 50), was purchased from Sinopharm Chemical Reagent Co., Ltd. (Shanghai, China), styrene (St, AR), sodium hydroxide (AR, ≥97.5%), sodium alginate (SA, 200–500 mp·s), and toluene (AR, ≥99.5%)was purchased from Adamas-beta Co., Ltd. (Shanghai, China), Shanghai. Deionized water was used throughout all experiments. All reagents were used as received without further purification.

### 4.2. Characterization Instruments

Microstructural photographs of the samples were taken using a scanning electron microscope (Hitachi, S-4800, Tokyo, Japan). The electrochemical performance of the hydrogels was evaluated using an electrochemical workstation (Chenhua, CHI-760E Shanghai, China). Universal mechanical testing machine (Shimazu, AGS-X, Dongguan, China) was utilized to perform the tensile tests. The diffraction spectra of the samples were determined by a fiber optic spectrometer (Ocean Optics, USB 4000-XR1-ES, Winter Park, FL, USA), with the reflectance spectra ranging between 400 and 800 nm.

### 4.3. Preparation Procedures

#### 4.3.1. Preparation of PVA/SA Hydrogels

An amount of 10 g of PVA was dissolved in deionized water at 90 °C to prepare a 20 wt% homogeneous PVA solution. Then, 1.2 g of SA was dissolved in deionized water at room temperature under stirring to prepare a 3 wt% SA solution. The PVA and SA solutions were mixed at different mass ratios and stirred thoroughly to obtain PVA/SA precursor solutions. The precursor solutions were poured into polytetrafluoroethylene molds, frozen at −20 °C for 24 h, and thawed at room temperature for 6 h to obtain physically crosslinked PVA/SA hydrogels.

#### 4.3.2. Preparation of Polystyrene and PC Templates

A 5 mL methacrylic acid (MA) and 200 mL of deionized water were added to a three-necked flask and heated to 98 °C in an oil bath. Then 80 mL of styrene (St, pretreated with NaOH to remove phenolic impurities) was added. After the solution reboiled, 5 mL of 9.7 wt% ammonium persulfate (APS) solution was introduced, and the mixture immediately turned milky white. After 20 min of reaction, a small portion of the emulsion was sampled, dropped onto a glass slide, and air-dried for color observation. Once the target color appeared, the flask was immediately transferred to an ice bath to quench the reaction. After cooling to room temperature, the synthesized polystyrene nanospheres were dialyzed in deionized water and stored for later use. Dialysis was performed using dialysis bag (MWCO 8000-14000, Sinopharm, Shanghai, China). The latex was dialyzed for at least 14 days, and deionized water was refreshed every 8 h to eliminate residuals and small-molecule impurities.

A certain amount of dialyzed polystyrene latex (typically 0.5 mL) was diluted 100-fold with deionized water. A glass slide was vertically immersed in the diluted latex and placed in a 70 °C oven. After complete evaporation of water, polystyrene nanospheres self-assembled into a highly ordered periodic array on the glass slide, exhibiting bright structural color, and was denoted as the PC template [22].

#### 4.3.3. Preparation of PVA/SA–PC

The glass slide carrying the PC template was tilted at 30°. A 1 mL syringe was used to slowly drop the PVA/SA precursor solution (~0.5 mL) onto the template. Under the action of gravity and capillary force, the precursor solution uniformly infiltrated the voids of the PC template. Another slide could be covered to form a chamber that contains the PVA/SA precursor solution and the PC template. The glass slides were pressed for 5 min to ensure the infiltration of precursor solution, and the excess solution was removed by tissue paper. The slide was then placed flat in a 70 °C oven for 10 h to cure into a film. The resulting PVA/SA–PC film was peeled off with tweezers and stored in a sealed bag for characterization.

### 4.4. Testing and Characterization

The as-prepared hydrogels were quenched in liquid nitrogen, freeze-dried for 48 h, sputter-coated with gold, and observed under SEM to characterize the microstructure.

For tensile tests, PVA/SA hydrogels were cut into standard dumbbell-shaped specimens with a gauge length of 40 mm, thickness of 2 mm, and width of 4 mm. Tensile testing was performed on a universal mechanical testing machine at room temperature with a crosshead speed of 50 mm/min until fracture. The maximum elongation and failure force were recorded. The strain ε and stress σ were calculated by Equations (2) and (3), respectively. Stress–strain curves were obtained, and each sample was tested in triplicate for stability.

(2) $$\varepsilon =\frac{L-L_{0}}{L_{0}}\times 100\%$$

where the *L* is the stretched length and *L*_0_ is the initial gauge length.

(3) $$\sigma =\frac{F}{S}$$

where the *F* is the applied tensile load and *S* is the cross-sectional area of the hydrogel sample.

A fiber optic spectrometer was used to measure the diffraction wavelength shift in PVA/SA–PC films.

### 4.5. Conductivity and Sensing Performance Tests

Hydrogel samples with uniform dimensions (20 mm × 20 mm × 2 mm) were prepared. A CHI-760E electrochemical workstation was used to measure AC impedance via the two-electrode method. The bulk resistance was determined from the intersection of the impedance curve with the real axis. The electrical conductivity (S/m) was calculated by Equation (4).

(4) $$\sigma =\frac{L}{RS}$$

where *L* is the sample length (m), *R* is the measured bulk resistance (Ω), and *S* is the cross-sectional area (m^2^) of the sample.

A standard 5 mm diameter green LED (generic commercially available component) was used, powered by two 1.5 V batteries connected in series; the LED bulb and batteries were connected to both ends of the film. The brightness variation in the bulb was observed during tensile deformation.

## Data Availability

The data supporting the findings of this manuscript are available from the corresponding authors upon reasonable request.
